# Pathologic Misclassification of Renal Cell Carcinoma in Adolescents and Young Adults

**DOI:** 10.3390/cancers18132020

**Published:** 2026-06-23

**Authors:** Megan Stout, Derek B. Allison, Leslie Peard, Will Cranford, Yana B. Feygin, Kara McAbee, Christopher J. McLouth, Patrick J. Hensley, Jason R. Bylund, Amanda F. Buchanan

**Affiliations:** 1 Department of Urology, Vanderbilt University, Nashville, TN 37232, USA; megan.stout@vumc.org; 2 Department of Pathology & Laboratory Medicine, University of Kentucky, Lexington, KY 40506, USA; 3 Department of Urology, University of Kentucky, Lexington, KY 40506, USA; 4 Department of Biostatistics, University of Kentucky, Lexington, KY 40506, USAyana.feygin@uky.edu (Y.B.F.);

**Keywords:** adolescent, young adult, renal cell carcinoma, kidney cancer, pathology, classification

## Abstract

**Background and Objective**: Diagnosis of TFE3-rearranged RCC (tRCC) requires a pathologist’s expertise and a high index of suspicion. The objectives of this study were to determine the rate of misclassification of RCC in the adolescent and young adult (AYA) patient population, identify any clinically significant impact associated with misclassification, and compare oncologic outcomes between patients with tRCC vs. other RCC subtypes. **Methods**: All patients < 50 years who underwent renal surgery and were diagnosed with renal cell carcinoma at a single institution from 2004 to 2019 were identified retrospectively. Pathology specimens were reviewed by a genitourinary pathologist. Changes in a pathologic diagnosis were denoted as “misclassified.” Clinical and oncologic data were analyzed comparing misclassified and non-misclassified groups as well as tRCC and other RCC diagnoses. A survival analysis was used to compare oncologic outcomes. **Results**: In total, 169 patients were identified, and 20/169 (11.8%) patients were misclassified after pathologic review, with 50% (*n* = 84) of this cohort < 40 years of age at diagnosis. There were significant differences between misclassified and non-misclassified groups with respect to race (*p* = 0.002), Appalachian county status (*p* = 0.035), and initial RCC subtype (*p* = 0.004). There were no significant differences in clinical or oncologic outcomes between tRCC and other RCC groups. There were no significant differences in time to death or recurrence in misclassified vs. non-misclassified patients (*p* = 0.83 and 0.68, respectively) or between patients with tRCC and other RCC subtypes (*p* = 0.45 and 0.062, respectively). **Conclusions**: >10% of patients with RCC were misclassified on the original pathologic diagnosis; however, there was no significant impact on oncologic outcomes. This identifies diagnostic blind spots for tRCC diagnosis in a young cohort. Perhaps reflex IHC or molecular testing could be of importance upfront for the AYA population with renal masses.

## Simple Summary

This project evaluated the rate of misclassification of renal cell carcinoma in young adult patients. Translocation renal cell carcinoma is the most common subtype in this age group but is relatively new and difficult to diagnose without a high index of suspicion. The misclassification rate of renal cell carcinoma is >10%, but this does not appear to affect oncologic outcomes.

## 1. Introduction

In 2004, the World Health Organization (WHO) first recognized translocation renal cell carcinoma (tRCC) as a distinct subgroup classification of renal cell carcinomas [1,2,3,4]. tRCC harbors a translocation involving either transcription factor E3 or EB (TFE3 and TFEB), both members of the microphthalmia (MiT) transcription factor family, resulting in a disruption of transcription regulation. The recent literature suggests that tRCC comprises 15% of RCC in patients ages 18–45 years (y) and 50–70% of all RCC in patients < 30 y, making it the most common type of RCC in children, adolescents, and young adults [5,6,7]. This observation is in contrast to the typical clear cell (ccRCC) pathology seen in older adults [2,3,4,5]. In general, tRCC is symptomatic in children, who present with a palpable abdominal mass, hematuria, or abdominal pain, while it is usually discovered incidentally in adults [8]. tRCC typically presents with more advanced disease. Ellati et al. found that >1/3 of tRCC patients presented with N1 disease, >50% presented with stage III disease or higher, and males were more likely to have metastatic disease [9,10].

tRCC lacks distinctive radiographic features from other renal tumors, making pathologic diagnosis critical [8]. The diagnosis, however, is challenging, and the true incidence is likely underestimated. On gross examination, tRCC appears similar to ccRCC, with a golden yellow color interspersed with varying degrees of necrosis, hemorrhage, and/or calcification. Histologically, it is similar to both papillary and ccRCC, with a nested/papillary architecture of clear cells and cells with granular eosinophilic cytoplasm [4]. The tumor stains inconsistently, and advanced molecular, chromosomal, and/or polymerase chain reaction (PCR) testing is not universally available [10].

The International Society of Urologic Pathology Vancouver reported that the most sensitive and specific method to detect tRCC is immunohistochemical (IHC) staining [11]. Over the past decade, as testing has become more widespread, the sensitivity and specificity of TFE3 IHC are variable depending on the antibody clone utilized and preanalytical factors, such as tissue fixation [12]. Additionally, increased TFE3 protein expression unrelated to fusion status can occur in a small subset of ccRCC, necessitating confirmatory testing with FISH or next-generation sequencing when the morphology is ambiguous [13]. Taken together, current practice often involves an algorithmic approach, utilizing a panel of IHC stains guided by the pretest probability of tRCC, which is informed by factors such as patient age and tumor morphology [14,15]. A high index of suspicion and a certain level of experience, such as individuals specializing in genitourinary pathology, are required to make the correct diagnosis. The authors suspect that a combination of all of these factors contributes to the suspected overall underreporting of the disease [4].

The objective of this study was to determine the incidence of misclassification of renal cell carcinoma (RCC) in the AYA patient population (age < 40 y), to compare this population with patients aged 40–49 y, and to assess for any potential clinical impact of misdiagnosis. The secondary objective was to compare outcomes between young patients with tRCC and those with other RCC diagnoses.

## 2. Materials and Methods

### 2.1. Study Population

All patients < 50 y that underwent renal surgery (radical or partial nephrectomy) for a renal mass from 1 January 2004 to 31 December 2019 at a tertiary care institution were included. Eligible patients had a pathologic diagnosis of RCC recorded in the medical record, with pathology slides available for review. Exclusion criteria included patients > 50 y, non-RCC diagnoses, and those who underwent cryoablation treatment or renal biopsy only. Any patients who underwent surgical intervention outside of the tertiary care institution or those with missing/unavailable slides for review were excluded.

### 2.2. Study Design

A retrospective chart review was performed to obtain baseline clinical data, patient demographics, including age at diagnosis, and the original pathologic diagnosis. Treatment details, including operative details or findings, as well as use of neoadjuvant or adjuvant therapies, were recorded. The Kentucky Cancer Registry was utilized to obtain oncologic outcome data for all patients meeting inclusion criteria, including recurrence records, details of adjuvant therapy, and mortality [16].

### 2.3. Pathology Review

Pathology slides were obtained from the pathology archives based on the previously noted inclusion and exclusion criteria. Pathology slides were originally classified historically at the time of the patient’s original renal surgery. Slides were then prospectively reviewed by a single pathologist with sub-specialization in genitourinary pathology via a standard algorithmic approach. The pathologist was blinded from any clinical information and the original diagnosis. All cases were originally reviewed by pathologists without specific genitourinary specialty training, and the expert genitourinary pathologist had not been involved with any of these cases. Histology slides were individually reviewed to confirm the diagnosis, provide a grade based on the current WHO/ISUP grading system, and assess for morphologic features commonly present in tRCC [4]. When appropriate, immunohistochemical (IHC) stains were performed to further classify the carcinomas, including CK7 (OV-TL 12/30 clone, DAKO Omnis, Agilent, Santa Clara, CA, USA), CA-IX (EP161 clone, Ventana, Roche, Indianapolis, IN, USA), EMA (E29 clone, DAKO Omnis, Agilent, Santa Clara, CA, USA), Cathepsin-K (3F9 clone, Abcam, Waltham, MA, USA), Fumarate Hydratase (J-13 clone, Santa Cruz, TX, USA), and TFE-3 (345R-18 clone, Cell Marque, Rocklin, CA, USA). All IHC was visualized with DAB. IHC stains were chosen based on pathologic interpretation and rationale for utilization based on established standards (Table 1) [15]. Furthermore, due to the fact that cases in the study set were identified prior to the 2022 WHO reclassification of “clear cell papillary renal cell carcinoma” to “clear cell papillary renal cell tumor”, these cases were included in the review [17].

### 2.4. Data Analysis

Demographics were reported utilizing parametric measures. Clinical characteristics were compared to the pathology review. A patient was designated as “misclassified” if the original histopathologic diagnosis was different from expert review. Comparisons between two groups (i.e., misclassified vs. not misclassified) were made utilizing the Wilcoxon rank sum test for continuous measures and Pearson’s Chi-squared or Fisher’s exact tests for categorical measures. Oncologic outcomes and survival comparisons between groups were made using Kaplan–Meier curves and log-rank testing. Statistical significance was determined with two-sided *p*-values < 0.05. All analyses were completed using R v4.4.2 [18].

## 3. Results

### 3.1. Clinical Demographics

A total of 169 patients met the inclusion criteria. Median age at diagnosis was 40 y (IQR [interquartile range] 34–43 y). One patient was <18 years of age (13 y). In total, 83 patients (49%) were aged 18–39 years, while 85 patients (50%) were aged 40–49 years of age. Median follow-up was 3 y (IQR 3–10 y).

### 3.2. Misclassification

**Figure A1:**
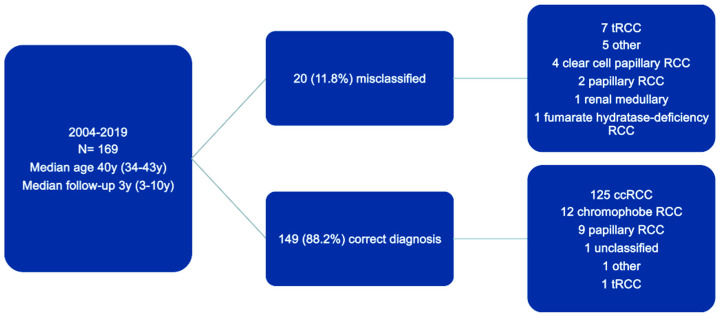
Patient population and misclassification summary.

Overall, 20/169 (11.8%) patients were misclassified after pathologic review, while 149/169 (88.2%) had the correct diagnosis (Figure A1). Seven (3.5%) of the 20 misclassified patients were re-classified as consistent with tRCC after expert review. All of these reclassified tumors showed the conventional staining patterns seen in Table 1. Other re-classified pathologic specimens upon expert review included clear cell papillary RCC (*n* = 4), papillary RCC (*n* = 2), renal medullary carcinoma (*n* = 1), fumarate hydratase-deficient RCC (*n* = 1), and RCC, not otherwise specified (NOS) (*n* = 5) (Table A1). In total, 2/20 patients were misclassified to a different subtype of papillary RCC, which could be considered a minor misclassification. Only one tRCC patient was correctly classified at initial diagnosis. When stratified by age, 50% of the misclassified cases were 18–39 y (*n* = 10), while the other 50% of misclassified cases were in the 40–49 y (*n* = 10).

No significant differences were observed between misclassified and non-misclassified patients in terms of age at diagnosis, clinical stage at diagnosis, adjuvant therapy received, pathologic nodal status, recurrence, or mortality (Table 2).

The year of diagnosis did not differ between groups. Notably, black patients (*p* = 0.002), individuals not from an Appalachian county of Kentucky (*p* = 0.035), and clear cell and papillary subtype RCC on initial review (*p* = 0.004) were more likely to be misclassified. There were no differences in clinical or cancer-specific outcomes found between those with tRCC diagnoses compared to those with other RCC subtypes (Table 3).

### 3.3. Oncologic Outcomes

**Figure 1:**
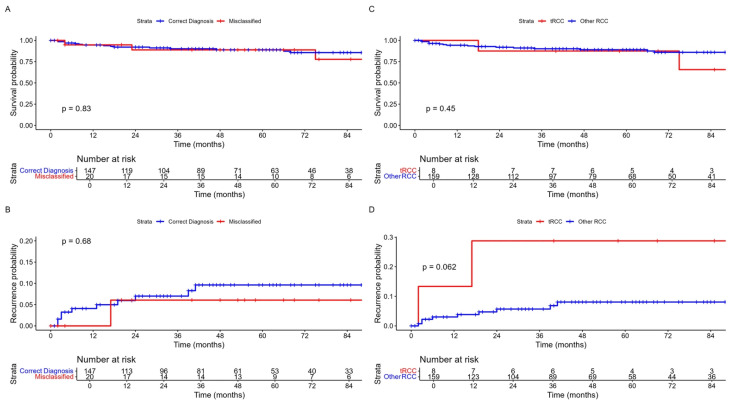
Survival probability (**A**,**C**) and recurrence (**B**,**D**) probability stratified by correct diagnosis versus misclassification (**A**,**B**) and RCC subtype (**C**,**D**).

When comparing the misclassified and non-misclassified groups, there were no significant differences in time to death (*p* = 0.83, 5-year survival 88.8% vs. 88.9%) or time to recurrence (*p* = 0.68, 5-year non-recurrence 93.8% vs. 90.4%) (Figure 1). The tRCC and other tRCC groups also did not have different times to death (*p* = 0.45, 5-year survival 87.5% vs. 89.0%) or time to recurrence (*p* = 0.062, 5-year non-recurrence 75.0% vs. 91.9%).

## 4. Discussion

Of the 169 patients identified, 20 patients (11.8%) were misclassified after expert pathologic review, with 51% (*n* = 10) of these cases identified in the <40 years of age cohort. Seven (3.5%) of these patients were re-classified as consistent with tRCC, which was the largest RCC subtype in the misclassified group. Interestingly, only one patient with tRCC was accurately diagnosed on the original pathology. There were significant differences between misclassified and non-misclassified groups in terms of race (*p* = 0.002), Appalachian county status (*p* = 0.035), and initial RCC subtype (*p* = 0.004). No other clinical features, such as age or year of diagnosis, were statistically significant. Oncologic outcomes did not differ based on misclassification status or by RCC subtype. The results of this study contribute to the description of RCC misclassification rates in AYA populations and suggest that misclassification may not impact cancer-specific outcomes.

The reported incidence of tRCC in the adult population varies substantially in the literature from 0.9 to 9%, compared to as high as 50–70% of all RCC in patients < 30 y [19,20,21,22,23,24]. This wide range in incidence may be secondary to underdiagnosis or misclassification of tRCC, as described previously [19,20,21,22,23,24]. Discrepancy in pathologic diagnostic interpretation is not uncommon, with incidence across all fields of medicine ranging anywhere from 11 to 40% [25,26,27]. In most instances, differences in diagnosis are minor with little to no clinical consequence. However, a major diagnostic discordance could have clinically significant long-term prognostic management and treatment implications, and possibly even alter patient outcomes. For example, Peck et al. compared specimens with a major discordance. The rate of inaccurate diagnoses ranged from 3 to 9% among specimen groups (including nine urology cases out of 80 cases reviewed), with the highest rates of inaccurate diagnoses in gynecologic, dermatopathologic, and gastrointestinal specimens [28]. Diagnostic discrepancies in surgical pathology could have significant implications for patient care. Johnson et al. evaluated 740 s opinion pathology cases following referral to a tertiary center, where 14.1% of the cases had diagnostic discrepancy, with 4.1% of these cases resulting in a change in care [29]. Inconsistency in identifying and communicating errors in pathologic diagnosis remains a concern, and there is no “appropriate” rate of misclassification widely accepted among pathologists or institutions [30].

There are studies that describe factors associated with correct pathologic diagnosis, including prostate cancer [31,32]. Pathology discordance or disagreement ranges between 10 and 15% depending on primary organ site, specimen type obtained, and the nature of the discrepancy [33]. In the realm of renal malignancy, much of the literature focuses on ccRCC. Pathologic validation was performed of renal cell carcinoma histology in the Surveillance, Epidemiology, and End Results (SEER) program, which confirmed that 98.5% of specimens were indeed RCC. The positive predictive value was higher for ccRCC than other subtypes, suggesting that confirmation may be warranted for cases of non-ccRCC [30]. Recognizing tRCC can be challenging, as it may be virtually indistinguishable from other RCC subtypes by gross examination alone; histopathologic examination by an expert eye may be critical to accurate diagnosis, especially in settings of unusual cell pattern or limited tissue availability [31]. Identifying a pathologist with genitourinary-focused experience at high-volume RCC centers may aid in the detection of critical features and help prevent misclassification. Taylor et al. published their 7-year findings of a urological second opinion consult service to improve pathologists’ awareness of diagnosis discrepancies. They found an overall mean disagreement rate of 15.2% across all GU specimens, with kidney specimens having a 15.4% disagreement rate on second opinion review [33]. Another multi-specialty cancer center study reported a 6.7% discordance rate specifically for GU pathologic specimens on second opinion review. Surprisingly, the highest rates of major discordance that led to clinically significant management changes were only found in 1% of cases [34]. Similarly, in this study, we found a similar percentage of this cohort with misclassification status after pathologic re-review (11.8%). The present findings of no significant differences in clinical outcomes such as time to death or recurrence between misclassified vs. non-misclassified groups can be inferred that, similarly, rarely do these misclassifications lead to clinically significant management changes.

When comparing misclassified and non-misclassified groups within this patient cohort, there were no significant differences in groups in regard to age, sex, year of surgical diagnosis, surgical approach, nodal status, surgical stage, or exposure to adjuvant or neoadjuvant therapies. There were differences, however, with respect to race, Appalachian county status, and initial RCC subtype. Black patients and those from non-Appalachian counties were more likely to be misclassified. It is unclear if this finding is related to the pathologists’ exposure at the institution (which serves predominantly white patients from Appalachian counties) or if this would be seen across multiple institutions. Due to the limited numbers in this experience and the underpowered nature of the comparison, this study is exploratory only and hypothesis-generating. Clear cell and papillary subtypes of RCC on initial diagnosis were also more likely to be misclassified on initial review. This is intuitive since these subtypes have a similar histologic architecture to tRCC, which inevitably was found to be the most common re-classified subtype on expert pathologist review [4].

No contemporary studies describe tRCC misclassification rates specifically. RCCs are relatively rare in children and young adults, and though specimens may share many histological similarities to typical RCC occurring in adulthood, there remain unique clinico-pathological differences [32]. The rate of tRCC has been reported to be as high as 41.5% in the pediatric and young adult patient populations. The implications, however, of misclassification of tRCC on oncologic management and outcomes, remain unknown [8]. There are novel trials such as NORDIC-SUN and AREN1721 exploring the role of immune checkpoint and tyrosine kinase inhibition in the treatment of tRCC that may have a future impact on the treatment of AYA patients [35]. Therefore, the importance of the correct diagnosis of tRCC can help both patients and researchers alike. In the present study, it is encouraging that there were no differences in recurrence or cancer-specific mortality based on misclassification. While it appears that misclassification does not impact oncologic outcomes, the authors caution that the present study is limited by sample size and warrants further evaluation across larger groups.

The clinical behavior of tRCC may vary between age groups, with the emerging literature suggesting that the pediatric and adult forms of tRCC may be distinct in biology and clinical behavior, prompting different treatment protocols [3,19,36,37]. In children, tRCC may have a more indolent course and a favorable prognosis [11]. In a large meta-analysis of pediatric tRCC patients following surgical resection, 93% of patients with N1M0 disease were disease-free at a mean follow-up of 6.3 y, with most (89%) not receiving any adjuvant therapy [3]. In contrast, in the adults with tRCC, there are reports of a rapidly progressive disease with an average survival of 18 months from diagnosis [23,36]. Komai et al. reported that 40% of adults with stage III or IV tRCC died within 1 y of presentation. Interestingly, the specific gene fusion TFE3 was found to be exclusive to the patients who died [6]. Few studies have compared recurrence and mortality rates of tRCC versus other non-tRCCs [19]. The present cohort identified only one patient < 18 y and 17 patients < 30 y, limiting overall comparisons between the pediatric and AYA populations specifically. Further characterization of the natural history of tRCC is needed within individual age cohorts to better elucidate any differences that may exist.

### Limitations

This study has inherent limitations. The small sample size of misclassified patients and those with tRCC makes comparisons between subgroups difficult. No doubt the study is underpowered to detect any differences. A single subspecialty-trained GU pathologist reviewed all specimens, and this potentially introduces bias. However, efforts were made to limit this bias by blinding the pathologist to all clinical details. In the methodologic process, the study team believed it was more critical to have a single expert GU pathologist reviewer for specimen classification rather than several non-GU-focused pathologists, but this is a limitation. Additionally, the diagnosis of tRCC was made on immunohistochemistry alone, without molecular confirmation. While reliable, this may leave some residual uncertainty in case classification.

The institutional database utilized to document cancer-specific diagnoses and outcomes was not designed with the intent to address this study’s specific objective of identifying misclassified RCC diagnoses or outcomes related to misclassification; as such, the analysis is secondary. The study design is also inherently limited by its retrospective design at a single institution with changing diagnostic criteria during the time period studied, which may have led to a misclassification that was minor and not suspected to be clinically significant. For example, four of the misclassified pathologic specimens were ultimately reclassified as clear cell papillary renal cell carcinoma (ccpRCC), three originally classified between 2004 and 2016, and one between 2016 and 2022. Notably, ccpRCC was not recognized as a distinct diagnostic entity by the WHO until 2016 and was subsequently renamed clear cell papillary renal cell tumor (ccpRCT) in 2022 [17]. As such, the designation of these cases as “misclassified” should be interpreted with caution, as earlier diagnoses occurred during periods when this entity had not yet been formally defined or widely recognized. Consequently, apparent misclassification in these earlier timeframes may instead reflect the evolving understanding and nomenclature of renal tumor pathology rather than true diagnostic error. Overall, there remains limited variation in current treatment and adjuvant therapy options for the translocation subtype of RCC.

Despite the limitations, this study contributes to the current understanding of tRCC, a subclass that is just one component of the histologic and biologic heterogeneity of RCC. The findings from this study highlight areas for future prospective, multi-institutional studies, particularly diagnostic accuracy and potential outcome differences due to misclassification, in pediatric, adolescent, and young adult patients.

## 5. Conclusions

After expert pathologic review of young patients with RCC, >10% were misclassified on original pathology, although oncologic outcomes were not significantly different. Furthermore, the small cohort of tRCC patients did not have significantly worse recurrence and mortality outcomes compared to those with non-tRCCs, though the small sample size is limiting. This study contributes to the identification of diagnostic blind spots for AYAs with renal masses. Perhaps routine ancillary workups with IHC or molecular testing could be of importance in this population.

## Figures and Tables

**Table 1:** Panel of immunohistochemical stains performed to further classify carcinomas.

Marker	Clear Cell RCC	Papillary RCC	Clear Cell Papillary RCC	Chromophobe RCC	TFE3 tRCC	FH-Deficient RCC	Renal Medullary Carcinoma
CK7	Usually negative or only focal staining	Diffuse strong membranous and cytoplasmic staining	Diffuse strong staining	Diffuse strong staining in most cases	Usually focal or negative. Diffuse staining argues against tRCC	Usually negative or focal, although patchy positivity may occur	Usually negative or focal, although variable positivity may occur
CA-IX	Diffuse circumferential (“box-like”) membranous staining characteristic of conventional clear cell RCC	Usually negative or focal staining	Characteristic “cup-like” basolateral staining pattern with luminal sparing	Typically negative	Variable or focal staining	Usually negative or focal staining	Typically negative
EMA	Diffuse membranous and cytoplasmic staining	Usually positive	Usually positive	Diffuse membranous positivity is common	Often reduced, focal, or absent compared with other RCC subtypes	Usually positive	Usually positive
Cathepsin K	Typically negative	Typically negative	Typically negative	Usually negative	Strong diffuse cytoplasmic staining favors tRCC, although the lack of staining does not exclude the diagnosis	Negative	Negative
**TFE3**	Negative, although rare, weak staining may occur	Negative	Negative	Negative	Diffuse moderate to strong nuclear staining is highly sensitive and specific for TFE3 tRCC; however, staining may be affected by fixation	Negative	Negative
INI-1 (SMARCB1)	Retained nuclear staining	Retained nuclear staining	Retained nuclear staining	Retained nuclear staining	Retained nuclear staining	Retained nuclear staining	Loss of nuclear staining is characteristic and highly supportive of renal medullary carcinoma
FH	Retained staining	Retained staining	Retained staining	Retained staining	Retained staining	Loss of FH staining supports FH-deficient RCC	Retained staining
Utility/Comments	CA-IX positivity with a lack of CK7 supports conventional clear cell RCC	Diffuse CK7 with absence of CA-IX positivity supports papillary RCC	Combined CK7 positivity with cup-like CA-IX staining is highly characteristic	Diffuse CK7 positivity with negative CA-IX supports chromophobe RCC	Cathepsin K and diffuse nuclear TFE3 staining with reduced EMA expression support the diagnosis	Loss of FH strongly supports FH-deficient RCC in the appropriate morphologic setting	Loss of INI-1 expression in the appropriate clinical and morphologic context strongly supports renal medullary carcinoma

**Table 2:** Summary table of clinical and diagnostic characteristics, overall and stratified by misclassification status.

Characteristic	Overall N = 169 ^1^	Correct N = 149	Misclassified N = 20	*p*-Value ^2^
Age at diagnosis, median (Q1, Q3)	40.0 (34.0, 43.0)	40.0 (34.0, 43.0)	40.0 (33.5, 43.0)	>0.9
Age category, n (%)				>0.9
<18 y	1 (0.6%)	1 (0.7%)	0 (0.0%)	
18–39 y	83 (49.1%)	73 (49.0%)	10 (50.0%)	
40–49 y	85 (50.3%)	75 (50.3%)	10 (50.0%)	
Age dichotomous, n (%)				>0.9
<40 y	84 (49.7%)	74 (49.7%)	10 (50.0%)	
40–49 y	85 (50.3%)	75 (50.3%)	10 (50.0%)	
Sex, n (%)				0.055
Female	68 (40.2%)	56 (37.6%)	12 (60.0%)	
Male	101 (59.8%)	93 (62.4%)	8 (40.0%)	
Race, n (%)				**0.002**
White	158 (94.0%)	143 (96.6%)	15 (75.0%)	
Black	10 (6.0%)	5 (3.4%)	5 (25.0%)	
Missing	1	1	0	
Marital status, n (%)				0.14
Single	55 (34.4%)	48 (34.0%)	7 (36.8%)	
Married	78 (48.8%)	72 (51.1%)	6 (31.6%)	
Divorced/separated	24 (15.0%)	19 (13.5%)	5 (26.3%)	
Widowed	3 (1.9%)	2 (1.4%)	1 (5.3%)	
Missing	9	8	1	
Insurance status, n (%)				0.7
Not insured	23 (14.0%)	20 (13.8%)	3 (15.8%)	
Medicaid	51 (31.1%)	46 (31.7%)	5 (26.3%)	
Medicare	16 (9.8%)	13 (9.0%)	3 (15.8%)	
Private	62 (37.8%)	56 (38.6%)	6 (31.6%)	
Insured, unknown type	12 (7.3%)	10 (6.9%)	2 (10.5%)	
Missing	5	4	1	
Tobacco use, n (%)				0.11
Never	56 (38.4%)	53 (40.8%)	3 (18.8%)	
Yes	90 (61.6%)	77 (59.2%)	13 (81.3%)	
Missing	23	19	4	
Family history of malignancy, n (%)	87 (56.9%)	77 (57.9%)	10 (50.0%)	0.5
Missing	16	16	0	
Population of home county, n (%)				0.087
Rural, not adjacent to Metro	27 (16.0%)	27 (18.1%)	0 (0.0%)	
Rural, adjacent to Metro	3 (1.8%)	3 (2.0%)	0 (0.0%)	
Urban 2500–19,999, not adjacent to metro	51 (30.2%)	45 (30.2%)	6 (30.0%)	
Urban 2500–19,999, adjacent to metro	17 (10.1%)	14 (9.4%)	3 (15.0%)	
Urban > 20,000, not adjacent to metro	2 (1.2%)	1 (0.7%)	1 (5.0%)	
Urban > 20,000, adjacent to metro	13 (7.7%)	12 (8.1%)	1 (5.0%)	
Metro area 250,000–1 M	54 (32.0%)	46 (30.9%)	8 (40.0%)	
Metro area > 1 M	2 (1.2%)	1 (0.7%)	1 (5.0%)	
Appalachian status, n (%)				**0.035**
Appalachian county	101 (59.8%)	94 (63.1%)	7 (35.0%)	
Not Appalachian county	66 (39.1%)	53 (35.6%)	13 (65.0%)	
Non-KY county	2 (1.2%)	2 (1.3%)	0 (0.0%)	
Cytotoxic chemo history, n (%)	5 (3.0%)	4 (2.7%)	1 (5.0%)	0.5
Missing	3	3	0	
Initial RCC, n (%)				**0.004**
Clear cell	136 (80.5%)	124 (83.2%)	12 (60.0%)	
Papillary type	13 (7.7%)	9 (6.0%)	4 (20.0%)	
Chromophobe	13 (7.7%)	12 (8.1%)	1 (5.0%)	
Translocation	1 (0.6%)	1 (0.7%)	0 (0.0%)	
Sarcomatoid differentiation	2 (1.2%)	1 (0.7%)	1 (5.0%)	
Unclassified	2 (1.2%)	2 (1.3%)	0 (0.0%)	
Other	2 (1.2%)	0 (0.0%)	2 (10.0%)	
Surgery year, n (%)				0.6
2008	13 (7.7%)	10 (6.7%)	3 (15.0%)	
2009	8 (4.7%)	7 (4.7%)	1 (5.0%)	
2010	17 (10.1%)	16 (10.7%)	1 (5.0%)	
2011	16 (9.5%)	14 (9.4%)	2 (10.0%)	
2012	16 (9.5%)	16 (10.7%)	0 (0.0%)	
2013	20 (11.8%)	17 (11.4%)	3 (15.0%)	
2014	17 (10.1%)	13 (8.7%)	4 (20.0%)	
2015	24 (14.2%)	22 (14.8%)	2 (10.0%)	
2016	15 (8.9%)	12 (8.1%)	3 (15.0%)	
2017	16 (9.5%)	15 (10.1%)	1 (5.0%)	
2018	6 (3.6%)	6 (4.0%)	0 (0.0%)	
2021	1 (0.6%)	1 (0.7%)	0 (0.0%)	
Overall stage, n (%)				0.6
I	127 (75.1%)	111 (74.5%)	16 (80.0%)	
II	11 (6.5%)	11 (7.4%)	0 (0.0%)	
III	30 (17.8%)	26 (17.4%)	4 (20.0%)	
IV	1 (0.6%)	1 (0.7%)	0 (0.0%)	
Surgery type, n (%)				0.6
Radical Nx	75 (44.4%)	65 (43.6%)	10 (50.0%)	
Partial Nx	94 (55.6%)	84 (56.4%)	10 (50.0%)	
Surgery approach, n (%)				0.2
Open	19 (11.2%)	18 (12.1%)	1 (5.0%)	
Lap	102 (60.4%)	86 (57.7%)	16 (80.0%)	
Robot	48 (28.4%)	45 (30.2%)	3 (15.0%)	
Node sampling performed, n (%)	31 (18.3%)	25 (16.8%)	6 (30.0%)	0.2
LN yield, median (Q1, Q3)	1.0 (1.0, 3.0)	2.0 (1.0, 4.0)	1.0 (1.0, 2.0)	0.4
Missing	140	126	14	
Follow up (months), median (Q1, Q3)	47.0 (18.0, 84.0)	43.0 (18.0, 84.0)	62.0 (31.5, 91.5)	0.2
Missing	2	2	0	
Adjuvant treatment, n (%)	18 (10.7%)	16 (10.7%)	2 (10.0%)	>0.9
Neoadjuvant treatment, n (%)	1 (0.6%)	1 (0.7%)	0 (0.0%)	>0.9
pT at surgery, n (%)				0.6
1	127 (75.1%)	111 (74.5%)	16 (80.0%)	
2	1 (0.6%)	1 (0.7%)	0 (0.0%)	
3	30 (17.8%)	26 (17.4%)	4 (20.0%)	
4	11 (6.5%)	11 (7.4%)	0 (0.0%)	
pN status, n (%)				0.074
0	159 (97.5%)	141 (98.6%)	18 (90.0%)	
1	4 (2.5%)	2 (1.4%)	2 (10.0%)	
Missing	6	6	0	
Recurrence, n (%)	11 (6.5%)	10 (6.8%)	1 (5.0%)	>0.9
Missing	1	1	0	
Death, n (%)	19 (11.3%)	16 (10.8%)	3 (15.0%)	0.7
Missing	1	1	0	

^1^ median (Q1, Q3); n (%). ^2^ Wilcoxon rank sum test; Fisher’s exact test; Pearson’s Chi-squared test. Bold: statistically significant values <0.05.

**Table 3:** Summary table of clinical and diagnostic characteristics, overall and stratified by tRCC diagnosis after expert review.

Characteristic	Overall N = 169 ^1^	Patients Without tRCC N = 161	Patients with tRCC N = 8	*p*-Value ^2^
Age at diagnosis, median (Q1, Q3)	40.0 (34.0, 43.0)	40.0 (34.0, 43.0)	40.0 (29.5, 44.5)	>0.9
Age category, n (%)				>0.9
<18	1 (0.6%)	1 (0.6%)	0 (0.0%)	
18–39	83 (49.1%)	79 (49.1%)	4 (50.0%)	
40–49	85 (50.3%)	81 (50.3%)	4 (50.0%)	
Age dichotomous, n (%)				>0.9
<40	84 (49.7%)	80 (49.7%)	4 (50.0%)	
40 and older	85 (50.3%)	81 (50.3%)	4 (50.0%)	
Sex, n (%)				0.062
Female	68 (40.2%)	62 (38.5%)	6 (75.0%)	
Male	101 (59.8%)	99 (61.5%)	2 (25.0%)	
Race, n (%)				0.4
White	158 (94.0%)	151 (94.4%)	7 (87.5%)	
Black	10 (6.0%)	9 (5.6%)	1 (12.5%)	
Missing	1	1	0	
Marital status, n (%)				0.1
Single	55 (34.4%)	52 (34.2%)	3 (37.5%)	
Married	78 (48.8%)	76 (50.0%)	2 (25.0%)	
Divorced/separated	24 (15.0%)	22 (14.5%)	2 (25.0%)	
Widowed	3 (1.9%)	2 (1.3%)	1 (12.5%)	
Missing	9	9	0	
Insurance status, n (%)				0.5
Not insured	23 (14.0%)	22 (14.1%)	1 (12.5%)	
Medicaid	51 (31.1%)	50 (32.1%)	1 (12.5%)	
Medicare	16 (9.8%)	16 (10.3%)	0 (0.0%)	
Private	62 (37.8%)	57 (36.5%)	5 (62.5%)	
Insured, unknown type	12 (7.3%)	11 (7.1%)	1 (12.5%)	
Missing	5	5	0	
Tobacco use, n (%)				0.2
Never	56 (38.4%)	52 (37.1%)	4 (66.7%)	
Yes	90 (61.6%)	88 (62.9%)	2 (33.3%)	
Missing	23	21	2	
Family history of malignancy, n (%)	87 (56.9%)	84 (57.9%)	3 (37.5%)	0.3
Missing	16	16	0	
Population of home county, n (%)				0.12
Rural, not adjacent to metro	27 (16.0%)	27 (16.8%)	0 (0.0%)	
Rural, adjacent to metro	3 (1.8%)	3 (1.9%)	0 (0.0%)	
Urban 2500–19,999, not adjacent to metro	51 (30.2%)	50 (31.1%)	1 (12.5%)	
Urban 2500–19,999, adjacent to metro	17 (10.1%)	16 (9.9%)	1 (12.5%)	
Urban > 20,000, not adjacent to metro	2 (1.2%)	1 (0.6%)	1 (12.5%)	
Urban > 20,000, adjacent to metro	13 (7.7%)	13 (8.1%)	0 (0.0%)	
Metro area 250,000–1 M	54 (32.0%)	49 (30.4%)	5 (62.5%)	
Metro area > 1 M	2 (1.2%)	2 (1.2%)	0 (0.0%)	
Appalachian status, n (%)				0.3
Appalachian county	101 (59.8%)	98 (60.9%)	3 (37.5%)	
Not Appalachian county	66 (39.1%)	61 (37.9%)	5 (62.5%)	
Non-KY county	2 (1.2%)	2 (1.2%)	0 (0.0%)	
Cytotoxic chemo history, n (%)	5 (3.0%)	4 (2.5%)	1 (12.5%)	0.2
Missing	3	3	0	
Initial RCC, n (%)				0.12
Clear cell	136 (80.5%)	130 (80.7%)	6 (75.0%)	
Papillary type	13 (7.7%)	12 (7.5%)	1 (12.5%)	
Chromophobe	13 (7.7%)	13 (8.1%)	0 (0.0%)	
Translocation	1 (0.6%)	0 (0.0%)	1 (12.5%)	
Sarcomatoid differentiation	2 (1.2%)	2 (1.2%)	0 (0.0%)	
Unclassified	2 (1.2%)	2 (1.2%)	0 (0.0%)	
Other	2 (1.2%)	2 (1.2%)	0 (0.0%)	
Surgery year, n (%)				0.5
2008	13 (7.7%)	11 (6.8%)	2 (25.0%)	
2009	8 (4.7%)	8 (5.0%)	0 (0.0%)	
2010	17 (10.1%)	17 (10.6%)	0 (0.0%)	
2011	16 (9.5%)	16 (9.9%)	0 (0.0%)	
2012	16 (9.5%)	16 (9.9%)	0 (0.0%)	
2013	20 (11.8%)	18 (11.2%)	2 (25.0%)	
2014	17 (10.1%)	15 (9.3%)	2 (25.0%)	
2015	24 (14.2%)	23 (14.3%)	1 (12.5%)	
2016	15 (8.9%)	15 (9.3%)	0 (0.0%)	
2017	16 (9.5%)	15 (9.3%)	1 (12.5%)	
2018	6 (3.6%)	6 (3.7%)	0 (0.0%)	
2021	1 (0.6%)	1 (0.6%)	0 (0.0%)	
Overall stage, n (%)				>0.9
I	127 (75.1%)	121 (74.7%)	6 (75.0%)	
II	11 (6.5%)	10 (6.2%)	1 (12.5%)	
III	30 (17.8%)	29 (17.9%)	1 (12.5%)	
IV	1 (0.6%)	1 (0.6%)	0 (0.0%)	
Surgery type, n (%)				>0.9
Radical Nx	75 (44.4%)	72 (44.7%)	3 (37.5%)	
Partial Nx	94 (55.6%)	89 (55.3%)	5 (62.5%)	
Surgery approach, n (%)				0.5
Open	19 (11.2%)	19 (11.8%)	0 (0.0%)	
Lap	102 (60.4%)	95 (59.0%)	7 (87.5%)	
Robot	48 (28.4%)	47 (29.2%)	1 (12.5%)	
Node sampling performed, n (%)	31 (18.3%)	29 (18.0%)	2 (25.0%)	0.6
LN Yield, median (Q1, Q3)	1.0 (1.0, 3.0)	2.0 (1.0, 4.0)	1.0 (1.0, 1.0)	0.2
Missing	140	134	6	
Follow up (months), median (Q1, Q3)	47.0 (18.0, 84.0)	45.0 (18.0, 84.0)	72.0 (49.0, 91.5)	0.2
Missing	2	2	0	
Adjuvant treatment, n (%)	18 (10.7%)	17 (10.6%)	1 (12.5%)	>0.9
Neoadjuvant treatment, n (%)	1 (0.6%)	1 (0.6%)	0 (0.0%)	>0.9
pT at surgery n (%)				>0.9
I	127 (75.1%)	121 (74.7%)	6 (75.0%)	
II	11 (6.5%)	10 (6.2%)	1 (12.5%)	
III	30 (17.8%)	29 (17.9%)	1 (12.5%)	
IV	1 (0.6%)	1 (0.6%)	0 (0.0%)	
N at surgery, n (%)				0.2
0	159 (97.5%)	152 (98.1%)	7 (87.5%)	
1	4 (2.5%)	3 (1.9%)	1 (12.5%)	
Missing	6	6	0	
Recurrence, n (%)	11 (6.5%)	9 (5.6%)	2 (25.0%)	0.088
Missing	1	1	0	
Death, n (%)	19 (11.3%)	17 (10.6%)	2 (25.0%)	0.2
Missing	1	1	0	

^1^ median (Q1, Q3); n (%). ^2^ Wilcoxon rank sum test and Fisher’s exact test.

## Data Availability

Data are unavailable due to privacy or ethical restrictions, but are available upon request.

## Abbreviations

The following abbreviations are used in this manuscript:

tRCC	translocation renal cell carcinoma
y	years
IQR	interquartile range
WHO	World Health Organization
TFE3 and TFEB	transcription factor E3 and EB
MiT	microphthalmia transcription
ccRCC	clear cell renal cell carcinoma
PCR	polymerase chain reaction
IHC	immunohistochemical
SEER	Surveillance, Epidemiology, and End Results

## Appendix A

**Table A1:** Summary of misclassified cases and final diagnosis following pathologic review with those < 40 y (gray) versus those 40–49 y (blue).

Original Diagnosis (Misclassified)	Correct Diagnosis
Clear cell	tRCC
Sarcomatoid differentiation	Renal medullary
Papillary type 1	Papillary type 1,2
Other (microcystic)	Fumarate-hydratase-deficient
Clear cell	tRCC
Clear cell	Other
Clear cell	Clear cell papillary
Clear cell	tRCC
Papillary type 1/2	Clear cell papillary
Other (multicystic)	Other
Clear cell	Clear cell papillary
Clear cell	Other
Clear cell	tRCC
Chromophobe	Other
Clear cell	Clear cell papillary
Clear cell	Other
Papillary type 2	tRCC
Papillary type 1	Papillary type 1,2
Clear cell	tRCC
Clear cell	tRCC
